# Type II Minocycline-Induced Hyperpigmentation During Long-Term Management of an Epidermal Growth Factor Receptor (EGFR) Inhibitor-Associated Acneiform Eruption

**DOI:** 10.7759/cureus.114957

**Published:** 2026-08-21

**Authors:** Dakarai Dunbar, Jacqueline Hwang, Marcia Driscoll

**Affiliations:** 1 Department of Dermatology, University of Maryland School of Medicine, Baltimore, USA

**Keywords:** chemotherapy-related toxicity, dermatology, drug-related side effects and adverse reactions, nephrology, oncology

## Abstract

Epidermal growth factor receptor (EGFR) inhibitors commonly cause papulopustular eruptions that may significantly impair quality of life and interfere with continuation of effective oncologic therapy. Oral tetracyclines remain the cornerstone of treatment because of their anti-inflammatory properties; however, prolonged therapy, particularly with minocycline, carries a risk of cumulative adverse effects, including drug-induced hyperpigmentation. We report the case of a 40-year-old man with hereditary leiomyomatosis and renal cell carcinoma (HLRCC) who developed an EGFR inhibitor-associated papulopustular eruption shortly after initiating erlotinib and bevacizumab. Persistent disease despite doxycycline therapy and concern for doxycycline-associated photosensitivity prompted a transition to long-term minocycline. After approximately 29 months of treatment, the patient developed symmetric slate-blue macules on the bilateral shins consistent with type II minocycline-induced hyperpigmentation. Reduction of the minocycline dose resulted in gradual improvement of the pigmentation while maintaining adequate control of the eruption. This case highlights the importance of longitudinal dermatologic follow-up in patients receiving prolonged tetracycline therapy for EGFR inhibitor-associated cutaneous toxicities. Periodic reassessment of treatment necessity, dose optimization, and early recognition of delayed medication-related adverse effects may minimize cumulative toxicity while preserving effective oncologic therapy.

## Introduction

Hereditary leiomyomatosis and renal cell carcinoma (HLRCC) is a rare autosomal dominant cancer syndrome caused by pathogenic variants in the fumarate hydratase gene. Patients with HLRCC are predisposed to an aggressive form of renal cell carcinoma and frequently require systemic targeted therapy. Combination treatment with bevacizumab and erlotinib, an epidermal growth factor receptor tyrosine-kinase inhibitor (EGFR-TKI), has demonstrated clinical activity in patients with advanced HLRCC-associated renal cell carcinoma [1].

Cutaneous toxicities are among the most common adverse effects of EGFR-TKI therapy. The most frequent manifestation is a papulopustular eruption, commonly referred to as an acneiform eruption, which typically develops within the first several weeks of treatment. EGFR blockade disrupts keratinocyte signaling and follicular homeostasis, resulting in impaired epidermal differentiation and a sterile inflammatory folliculitis involving seborrheic and sun-exposed skin [2]. Because these eruptions can adversely affect quality of life and potentially compromise adherence to effective oncologic therapy, appropriate dermatologic management is important to facilitate continuation of cancer treatment.

Systemic tetracyclines, including doxycycline and minocycline, are commonly used to manage EGFR inhibitor-associated papulopustular eruptions because of their anti-inflammatory properties. Although generally well tolerated, prolonged tetracycline therapy may result in treatment-limiting adverse effects. Minocycline-induced hyperpigmentation is a well-recognized complication of long-term therapy that may develop months to years after treatment initiation and presents in several distinct clinical patterns [3,4]. This creates a particular therapeutic challenge when minocycline is providing effective control of a chronic treatment-related eruption but continued exposure contributes to cumulative toxicity.

We present a case of type II minocycline-induced hyperpigmentation that developed after prolonged minocycline therapy for an EGFR inhibitor-associated papulopustular eruption in a patient with HLRCC. Although minocycline-induced hyperpigmentation and its occurrence during treatment of EGFR inhibitor-associated eruptions have previously been described, this case highlights the clinical challenge of managing cumulative toxicity when the causative medication remains effective for the underlying cutaneous adverse event. Rather than discontinuing minocycline entirely, dose reduction was followed by improvement in hyperpigmentation while maintaining control of the papulopustular eruption. This case illustrates an individualized approach to balancing treatment efficacy and cumulative toxicity and, in the context of evolving consensus recommendations, highlights the importance of considering alternative non-antibiotic strategies for patients requiring prolonged management of EGFR inhibitor-associated cutaneous adverse events.

## Case presentation

A 40-year-old man with HLRCC presented for dermatologic evaluation of a new pruritic eruption that developed shortly after initiating bevacizumab (1,200 mg) and erlotinib (100 mg daily) for metastatic renal cell carcinoma. One week after starting erlotinib, he developed numerous monomorphic inflammatory papules and pustules involving the forehead, nose, upper chest, and upper back (Figures 1A-1C). The eruption involved approximately 24% of the body surface area and was consistent with a moderate EGFR inhibitor-associated papulopustular eruption. His second dose of erlotinib was temporarily withheld by the oncology team, resulting in partial improvement before treatment was resumed. He denied fever, mucosal involvement, or other systemic symptoms. 

**Figure 1:**
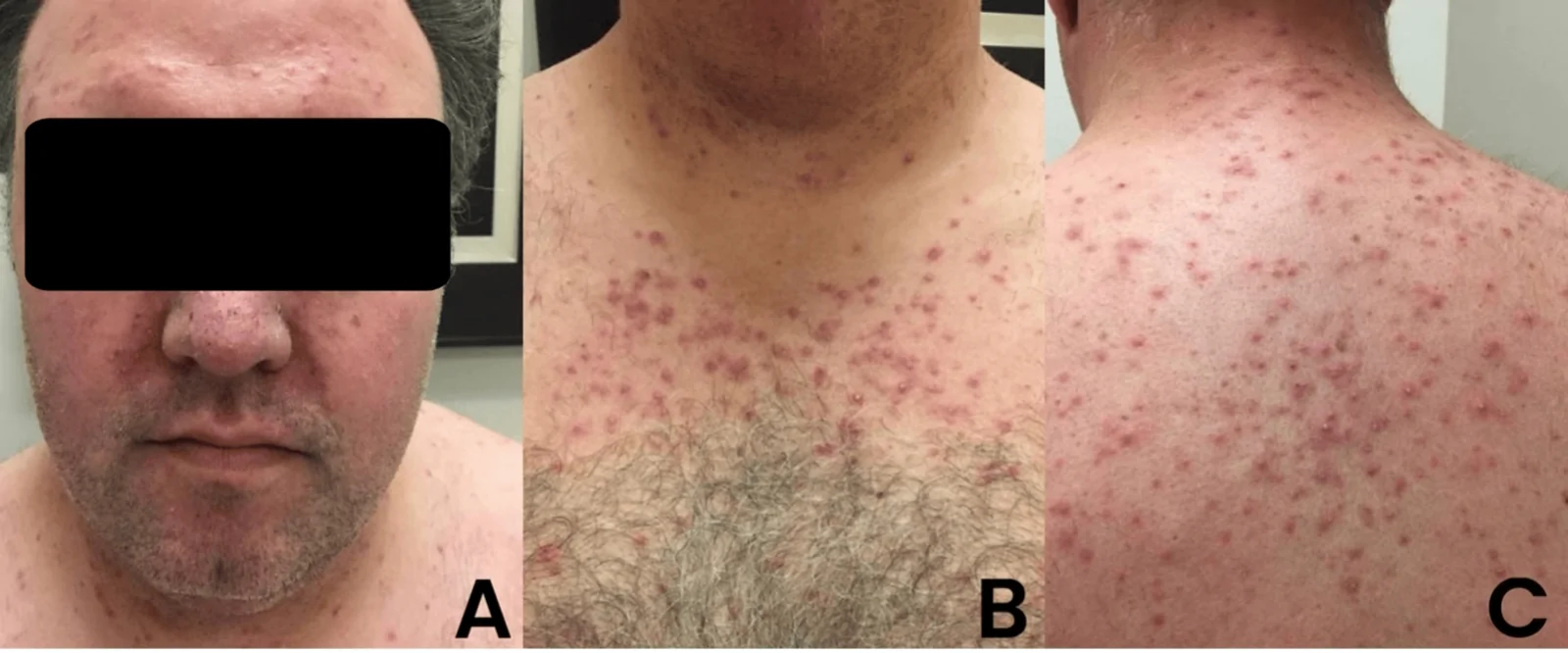
Numerous monomorphic pustules and papules with surrounding erythema are distributed on the face (A), upper chest (B), and upper back (C). The lesions are symmetric, non-scarring, and confined primarily to seborrheic and sun-exposed areas, consistent with an acneiform eruption.

Initial management included doxycycline 100 mg twice daily, clobetasol 0.05% cream applied to the chest and back twice daily for two weeks, triamcinolone 0.1% cream applied sparingly to affected areas of the face twice daily for two weeks, and hydroxyzine 10 mg three times daily as needed for pruritus. The topical corticosteroids were prescribed as short-course adjunctive anti-inflammatory therapy for the EGFR inhibitor-associated eruption rather than for acne vulgaris.

At four-month follow-up, the eruption remained incompletely controlled despite treatment. Because of persistent inflammatory disease and concern for doxycycline-associated photosensitivity related to the patient's frequent recreational sun exposure, doxycycline was transitioned to minocycline 100 mg twice daily. Topical clindamycin 1% lotion was added to the most affected areas, clobetasol 0.05% ointment replaced the cream formulation for truncal involvement, and the patient was counseled on photoprotection.

The eruption remained well controlled on minocycline therapy from December 2020 through May 2023 (approximately 29 months), during which the estimated cumulative dose exceeded 170 g. At follow-up, the patient reported the gradual onset of asymptomatic bluish-gray macules and patches involving both shins (Figures 2A, 2B). Physical examination demonstrated multiple well-demarcated, non-palpable, slate-blue to gray macules and patches symmetrically distributed over the bilateral anterior shins without associated erythema, scale, or induration. The clinical presentation was most consistent with type II minocycline-induced hyperpigmentation.

**Figure 2:**
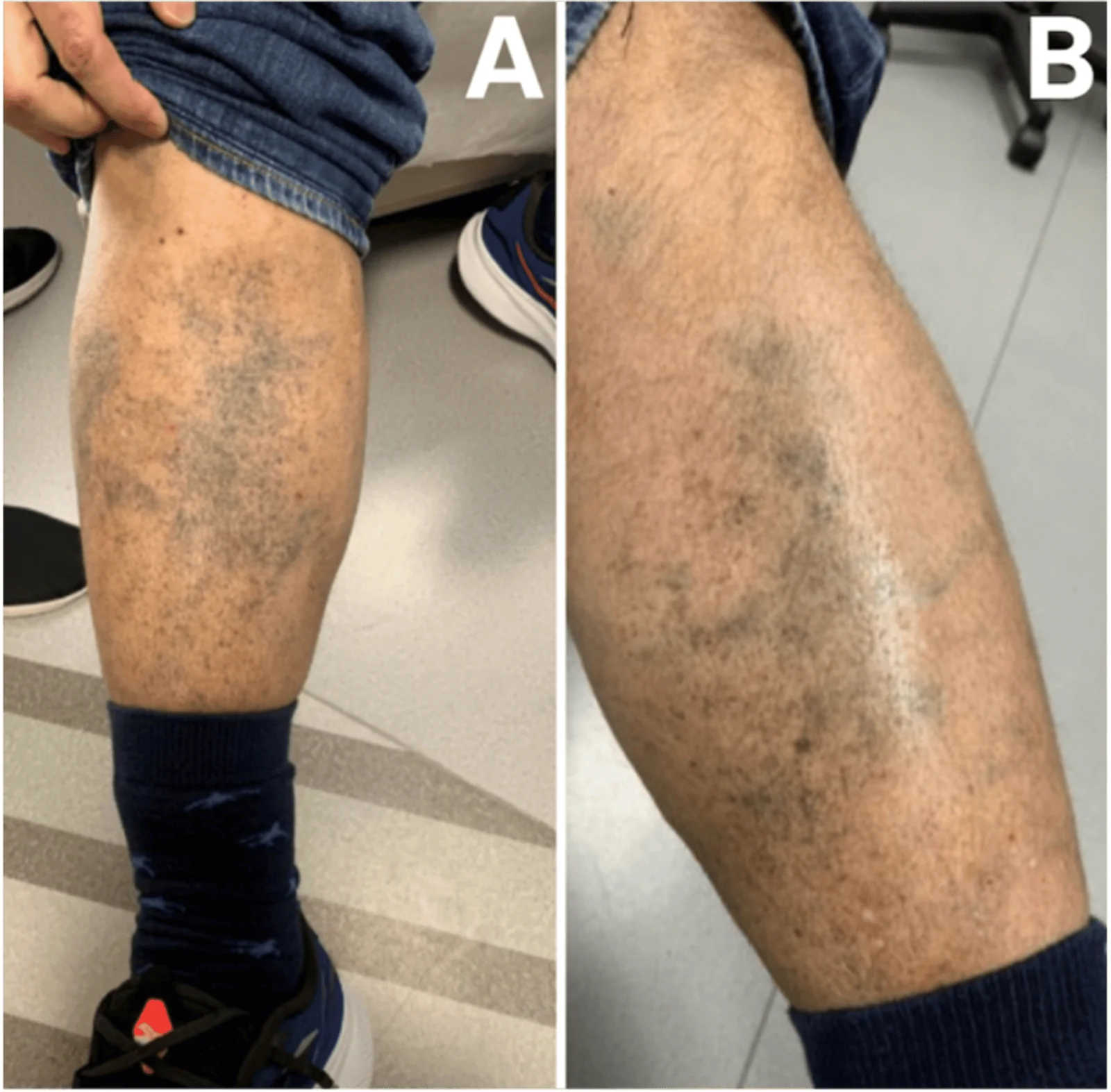
Multiple well-defined, slate-blue to gray macules and patches on the right (A) and left (B) shins. The lesions are nonpalpable and asymptomatic, with a symmetric distribution. No associated erythema, scaling, or induration is noted.

Skin biopsy was not performed because the characteristic clinical morphology, symmetric distribution, prolonged history of minocycline exposure, and absence of features suggestive of an alternative diagnosis were considered sufficient to establish the diagnosis, and histopathologic confirmation was unlikely to alter management. Laboratory evaluation was similarly deferred because there were no clinical findings to suggest another cause of acquired hyperpigmentation.

To minimize further cumulative drug exposure while maintaining control of the eruption, the minocycline dose was reduced to 100 mg daily. At two-month follow-up, partial improvement of the pigmentation was documented by the patient's oncologist, while the papulopustular eruption remained well controlled. Additional clinical photographs were unavailable because the patient elected to continue longitudinal dermatologic management through his oncology team.

## Discussion

Targeted therapies, including EGFR inhibitors, have significantly improved outcomes for patients with advanced malignancies but are frequently associated with cutaneous toxicities that can adversely affect quality of life and compromise adherence to life-prolonging therapy. Papulopustular eruptions are the most common dermatologic adverse events associated with EGFR inhibition and typically develop within the first several weeks of treatment. Unlike acne vulgaris, these eruptions result from disruption of EGFR signaling within the epidermis and pilosebaceous unit, leading to impaired keratinocyte proliferation, altered follicular differentiation, and sterile inflammatory folliculitis. Consequently, treatment focuses on controlling inflammation while preserving uninterrupted oncologic therapy whenever possible. Oral tetracyclines remain a cornerstone of systemic management because of their anti-inflammatory properties. Recent international Delphi consensus recommendations support doxycycline for systemic treatment and specifically identify minocycline as an alternative in patients with a history of photosensitivity or substantial ultraviolet exposure, consistent with the rationale for transitioning therapy in our patient [5]. Short courses of adjunctive topical corticosteroids may also be considered in selected patients with prominent inflammation. Although prolonged use of medium-potency topical corticosteroids on facial skin is generally discouraged, the triamcinolone prescribed in this case was intended as a brief adjunctive anti-inflammatory treatment rather than therapy for acne vulgaris [1-6].

Minocycline is frequently selected for the management of inflammatory dermatoses because of its anti-inflammatory properties; however, prolonged treatment increases the risk of cumulative medication-related adverse effects. Minocycline-induced hyperpigmentation is a well-recognized complication that has been reported in up to 15% of patients receiving long-term therapy and is generally associated with increasing duration and cumulative drug exposure. Clinically, four subtypes have been described (Table 1). Type I presents as blue-black pigmentation within areas of prior inflammation or scarring; Type II manifests as blue-gray pigmentation on previously normal skin, classically involving the shins and forearms; Type III appears as diffuse, muddy-brown pigmentation affecting sun-exposed skin; and the less commonly described Type IV resembles Type III but occurs within preexisting scars. Histologically, Type II pigmentation is characterized by dermal macrophages containing iron-chelated minocycline metabolites that stain positively with Perls Prussian blue and variably with Fontana-Masson stain [3,4].

**Table 1:** Clinical types of minocycline-induced hyperpigmentation. This table was independently created by the authors of this study based on the information obtained from Shute et al. [3]

Type	Clinical appearance	Typical distribution	Histopathologic findings	Clinical course
Type I	Blue-black macules or patches	Sites of prior inflammation or scarring	Iron-containing pigment within dermal macrophages (Perls positive)	Often improves after dose reduction or discontinuation
Type II	Blue-gray macules or patches	Previously normal skin (commonly shins or forearms)	Iron-chelated minocycline metabolites and melanin within dermal macrophages (Perls positive, variably Fontana-Masson positive)	May gradually improve after dose reduction or discontinuation
Type III	Diffuse muddy-brown pigmentation	Sun-exposed skin	Increased melanin within basal keratinocytes and dermal macrophages	Frequently persistent despite discontinuation
Type IV	Blue-gray or brown pigmentation within existing scars	Areas of previous scarring or inflammation	Histopathological features similar to Type III	Limited outcome data available

Our patient's presentation was most consistent with Type II minocycline-induced hyperpigmentation based on the characteristic slate-blue discoloration of the bilateral shins, prolonged minocycline exposure, and gradual improvement following dose reduction. Although skin biopsy may be useful when the diagnosis is uncertain or alternative causes of acquired dyspigmentation are strongly suspected, histopathologic confirmation was not pursued because the clinical morphology, medication history, and distribution were considered diagnostic. Biopsy findings would not have altered management [3].

Although minocycline-induced hyperpigmentation is a recognized adverse effect of chronic therapy, patients receiving EGFR inhibitors represent a unique population because treatment-related papulopustular eruptions may require prolonged systemic therapy to maintain symptom control while allowing continuation of effective targeted oncologic treatment. As a result, dermatologists must balance durable control of cutaneous toxicity against the cumulative risks associated with extended tetracycline exposure. In our patient, persistent disease despite doxycycline therapy and concern for doxycycline-associated photosensitivity prompted a transition to minocycline, which provided sustained disease control for more than two years before hyperpigmentation developed. Current consensus recommendations also provide a potential non-antibiotic alternative when tetracycline therapy is ineffective or contraindicated: low-dose isotretinoin (0.3 mg/kg) may be considered as second-line treatment for refractory grade II/III EGFR inhibitor-associated papulopustular eruptions [5]. In our patient, reduction of the minocycline dose allowed gradual improvement of the pigmentation while maintaining control of the eruption; however, alternative systemic therapy such as low-dose isotretinoin may be considered when adequate disease control cannot be maintained without continued tetracycline exposure. This case underscores the importance of longitudinal follow-up, periodic reassessment of treatment necessity, and individualized treatment modification to minimize medication-related toxicity while preserving effective management of EGFR inhibitor-associated cutaneous adverse events.

## Conclusions

In this case, persistent lesions despite doxycycline therapy prompted a transition to minocycline with adjunctive topical clindamycin and medium- to high-potency topical corticosteroid formulations. Minocycline effectively controlled the eruption over a prolonged period; however, chronic use ultimately resulted in cutaneous hyperpigmentation of the bilateral shins. Following dose reduction of minocycline, partial improvement in pigmentation was observed, highlighting the potential reversibility of this adverse effect and the importance of ongoing monitoring and dose adjustment in patients receiving prolonged tetracycline therapy.

This case highlights the importance of longitudinal dermatologic management in patients receiving epidermal growth factor receptor (EGFR) inhibitors, as cutaneous adverse effects may persist and evolve over extended treatment courses. Acneiform eruptions associated with EGFR inhibitors often require prolonged therapy, and while tetracycline-class antibiotics remain effective first-line agents, extended use - particularly of minocycline - carries a risk of delayed adverse effects such as drug-induced hyperpigmentation. Ongoing clinical reassessment is therefore essential to identify emerging complications and determine the lowest effective antibiotic dose. Individualized management strategies, including dose modification, antibiotic rotation, adjunctive topical therapies, and counseling on photoprotection, may help mitigate cumulative cutaneous toxicity. Proactive recognition and management of these delayed dermatologic sequelae can improve patient quality of life and help maintain adherence to targeted oncologic therapies through multidisciplinary care.
